# Expression of concern: Synthesis of a Fe_3_O_4_@P4VP@metal–organic framework core–shell structure and studies of its aerobic oxidation reactivity

**DOI:** 10.1039/d4ra90003k

**Published:** 2024-01-15

**Authors:** Zongcheng Miao, Xin Shu, Daniele Ramella

**Affiliations:** a Xijing University Xi'an Shaanxi Province 710123 P. R. China; b College of Science, Beijing University of Chemical Technology Beijing 100029 P. R. China shuxin@mail.buct.edu.cn; c Temple University-Beury Hall 1901, N. 13th Street Philadelphia PA 19122 USA

## Abstract

Expression of concern for ‘Synthesis of a Fe_3_O_4_@P4VP@metal–organic framework core–shell structure and studies of its aerobic oxidation reactivity’ by Zongcheng Miao *et al.*, *RSC Adv.*, 2017, **7**, 2773–2779, https://doi.org/10.1039/C6RA25820D.

The Royal Society of Chemistry is publishing this expression of concern in order to alert readers that concerns have been raised regarding the integrity of the XRD data in Fig. 2c and d.

The Royal Society of Chemistry has asked the affiliated institution (Beijing University of Chemical Technology) to investigate this matter and confirm the integrity and reliability of the XRD data in Fig. 2c and d.

An expression of concern will continue to be associated with this manuscript until we receive information from the institution on this matter.

Laura Fisher

9th January 2024

Executive Editor, *RSC Advances*

## Supplementary Material

